# A low-cost, multiplexable, automated flow cytometry procedure for the characterization of microbial stress dynamics in bioreactors

**DOI:** 10.1186/1475-2859-12-100

**Published:** 2013-10-31

**Authors:** Alison Brognaux, Shanshan Han, Søren J Sørensen, Frédéric Lebeau, Philippe Thonart, Frank Delvigne

**Affiliations:** 1 Unité de Bio-industries/CWBI, Gembloux Agro-Bio Tech, Université de Liège, Passage des Déportés 2, Gembloux 5030, Belgium; 2 Fond de la recherche scientifique (FRS-FNRS), Rue d’Egmont 5, Bruxelles 1000, Belgium; 3 Section for microbiology, Department of Biology, University of Copenhagen, Universitetsparken, 15, Bygning, 1, Copenhagen 2100, Denmark; 4 Unité de mécanique et construction, Gembloux Agro-Bio Tech, University of Liège, Passage des Déportés 2, Gembloux 5030, Belgium

**Keywords:** Automated flow cytometry, Phenotypic heterogeneity, *Escherichia coli*, Membrane permeability, Green fluorescent protein, Mini-bioreactors

## Abstract

**Background:**

Microbial cell population heterogeneity is now recognized as a major source of issues in the development and optimization of bioprocesses. Even if single cell technologies are available for the study of microbial population heterogeneity, only a few of these methods are available in order to study the dynamics of segregation directly in bioreactors. In this context, specific interfaces have been developed in order to connect a flow cytometer directly to a bioreactor for automated analyses. In this work, we propose a simplified version of such an interface and demonstrate its usefulness for multiplexed experiments.

**Results:**

A low-cost automated flow cytometer has been used in order to monitor the synthesis of a destabilized Green Fluorescent Protein (GFP) under the regulation of the *fis* promoter and propidium iodide (PI) uptake. The results obtained showed that the dynamics of GFP synthesis are complex and can be attributed to a complex set of biological parameters, i.e. on the one hand the release of protein into the extracellular medium and its uptake modifying the activity of the fis promoter, and on the other hand the stability of the GFP molecule itself, which can be attributed to the protease content and energy status of the cells. In this respect, multiplexed experiments have shown a correlation between heat shock and ATP content and the stability of the reporter molecule.

**Conclusion:**

This work demonstrates that a simplified version of on-line FC can be used at the process level or in a multiplexed version to investigate the dynamics of complex physiological mechanisms. In this respect, the determination of new on-line parameters derived from automated FC is of primary importance in order to fully integrate the power of FC in dedicated feedback control loops.

## Background

Clonal populations of microbial cells exhibit phenotypic heterogeneities due to environmental factors and even in homogenous environments, considering stochastic fluctuations at the level of biochemical reactions [1]. Microbial heterogeneity is governed by a complex set of intrinsic, extrinsic and external noise components that have been thoroughly studied at the fundamental level [2-4] but only partially applied in the field of bioprocess engineering [5]. Indeed, even though the heterogeneity of microbial populations has received a lot of attention over the past few years, the real impact of this phenomenon on microbial bioprocesses remains poorly understood. The main reason behind this lack of knowledge is the difficulty in monitoring microbial population heterogeneity in dynamic process conditions. In this respect, flow cytometry (FC) is a very powerful tool for following physiological properties of microbial cells under process-related conditions [6]. The main advantage of this method is to provide information about the phenotypic heterogeneity of a microbial population. This information is critical from a bioprocess improvement perspective since the appearance of an unwanted phenotype can impair its efficiency. Such phenomena have been recently been noted during culture of recombinant *Pichia pastoris* with the appearance of a non-secreting phenotype [7]. However, if many techniques are available for the determination of cell physiology at a given moment in a culture, dynamic evolution of microbial resistance to stress and adaptation is still poorly described. For this purpose, an automated version of FC has been proposed for on-line monitoring of cell population heterogeneity under process-related conditions [8,9]. Specific interfaces comprising a mixing chamber where microbial cells are diluted prior to analysis and stained (if required) have been developed and commercialized. In this way, FC could be used for feedback control at the level of a bioreactor control. Such application is of particular importance for the optimization of bioprocesses based on recombinant microorganism. Indeed, the induction of protein synthesis is often performed by adding a high concentration of inducer molecule at a given moment of the culture. However, this kind of protocol is known to induce a strong physiological stress at the level of the host cell which in turn impairs bioprocess productivity [10]. In this case, on-line FC can be used in order to monitor the intracellular protein synthesis at the single-cell level and the resulting signal can be exploited in order to trigger the feeding of an inducer. This kind of strategy has been previously investigated at the lab-scale level, but no practical applications are available at this time [11]. However, the use of automated FC is still largely underexploited in view of its power in the context of bioprocess optimization. No application of this technique for bioreactor control could be found, except for one very basic application where automated FC was used to control cell density inside a chemostat (cytostat) [12]. The main reason limiting the application of automated FC to microbial bioprocesses is the necessity for a complex interface between the FC and the bioreactor to be sampled. Commercial systems, such as the Flowcytoprep (MSP corp, MN) device, are available but are generally expensive [13]. In this work, we propose to use a benchtop Accuri flow cytometer as the basis for the design of an automated FC. This apparatus was recently tested on microbiological samples and led to reliable results [14]. In addition, fluid displacement is ensured by peristaltic pumps, facilitating the set-up of an interface with a bioreactor since no pressurization of the sample is needed. The development of previous systems was indeed impaired by the need to maintain pressure at the level of the sample unit [15-17]. Under this condition, FC can be easily interfaced to a bioreactor by using additional peristaltic pumps operated by a microcontroller. Sample dilution and staining is carried out in line in the tubing between the FC and the bioreactor. This automated FC system was tested by following the dynamics of an *Escherichia coli pfis::gfpAAV* fluorescent bio-reporter [18]. The reporter system consisted of an *E.coli* strain carrying a growth dependent promoter, in this case the *fis* promoter, fused to a gene expressing an unstable variant of GFP. The *pfis* promoter is induced in early stationary phase or when cells are shifted from low to high substrate availability [19]. This reporter system is thus a good indicator of the nutrient status of the cells. Indeed, in a mixing-deficient bioreactor, zones with high and low nutrient availability can coexist and significantly affect microbial physiology [20]. In order to increase the responsiveness of the reporter system, a destabilized *gfpAAV* variant exhibiting a half-life of less one hour was used [21]. The use of automated FC is thus particularly useful for this application since off-line sampling would affect the quality of the results. The combined use of the *pfis::gfpAAV* bio-reporter with automated FC was investigated for the first time. In a second study, the automated FC interface was multiplexed in order to monitor a platform of parallelized bioreactors. The high-throughput effectiveness of multiplexed FC was demonstrated by extracting dynamic data, giving new insights about the behaviour of the *gfpAAV* molecule under bioprocess conditions.

## Results and discussion

### Following *fis::gfpAAV* activity by automated FC

**Figure 1:**
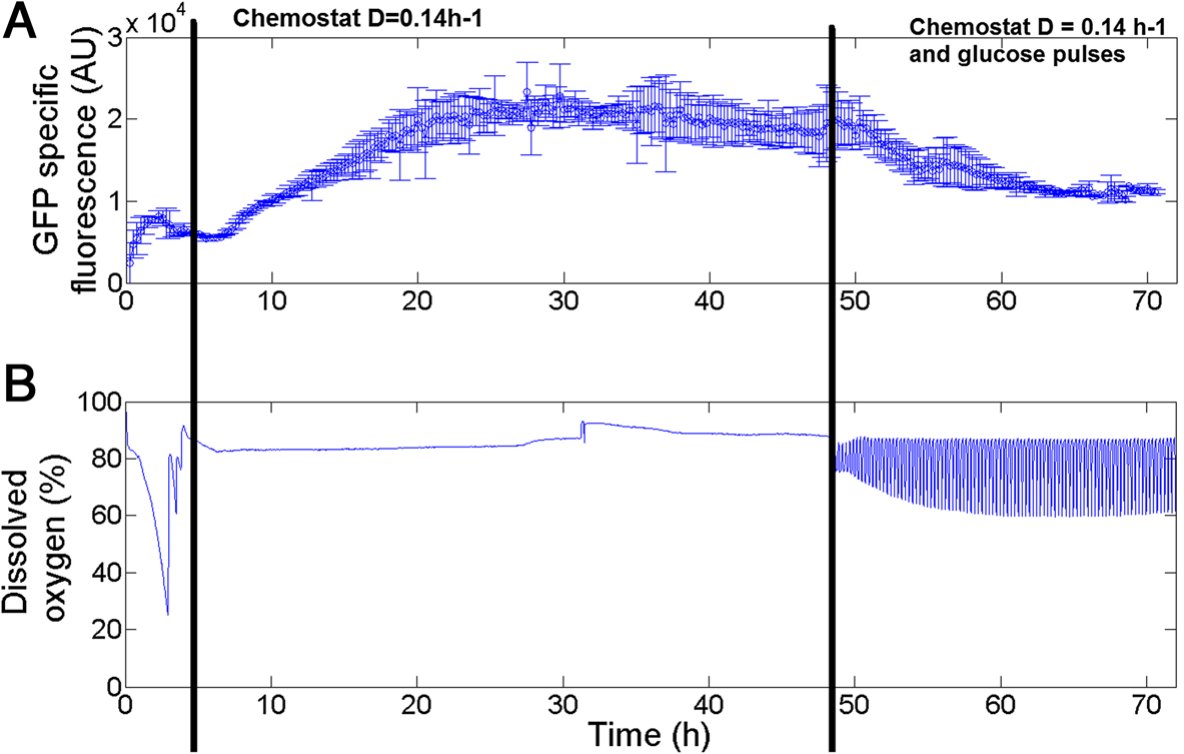
**Follow-up of the cultivation of *E. coli pfis::gfpAAV *in a 2 L stirred bioreactor by automated FC. A**: Monitoring GFP synthesis under the control of the *fis* promoter by automated FC. Each point corresponds to the mean fluorescence recorded after analysing 40,000 microbial cells **B**: The dissolved oxygen profile is shown in order to highlight the three distinct phases: batch, chemostat mode at D = 0.14 h^-1^ and chemostat mode at D = 0.14 h^-1^ with glucose pulses. The error bars displayed for the mean GFP fluorescence stand for two independent replicates of the cultivation.

In order to assess the efficiency of the automated FC platform, a first test was carried out on the basis of our previous results. Indeed, we have previously shown that the *fis::gfpAAV* system is strongly sensitive to substrate limitation and fluctuations [18], suggesting that this bio-reporter could be used to detect substrate concentration gradients in heterogeneous bioreactors. In order to assess this sensitivity, a chemostat experiment followed by a series of glucose pulses was conducted and the response of the GFP bio-reporter was followed by automated FC (Figure 1A). After an initial batch phase, a chemostat mode was set up at a dilution rate (D) of 0.14 h^-1^. After 43 hours of stabilization (corresponding to six residence times), glucose pulses were delivered at a given frequency. This latter phase can be visualized on the basis of the dissolved oxygen profile (Figure 1B), which fluctuates according to the glucose pulses. During the batch phase, GFP synthesis could be directly correlated to the growth rate by a relationship involving the square of the growth rate and known as the “mu square” rule [22]. This observation has previously been made with off-line GFP samples [18] and could be more efficiently validated with a greater number of sampling points using the automated FC. When the system is shifted to the chemostat mode, the GFP level is stable for a very short period of time (around 2 hours) and then rises. This phenomenon was unexpected since GFP would remain in steady-state if the activation of the *fis* promoter is effectively proportional to the growth rate, suggesting that additional physiological mechanisms are involved. In our previous studies, we have shown that proteins are secreted to the extracellular medium when microbial cells are exposed to prolonged substrate limitation [23,24]. This phenomenon has been reported by several authors and is generally termed protein leakage [25,26]. Since the *fis* promoter is known to be up-regulated when cells are shifted from minimal to rich medium conditions, this release of proteins could possibly contribute to an increase in the pool of available amino acids (a.a.) and thus turn the medium into a “rich medium” [19,27].

A comparison between off-line and on-line samples was performed and no significant differences were observed at the level of GFP synthesis (Additional file 1: Figure S1). Moreover, off-line analyses of extracellular proteins in supernatants confirmed the occurrence of protein leakage during the chemostat phase at D = 0.14 h^-1^ (Additional file 2: Figure S2), potentially explaining the rise in GFP synthesis during this phase according to our hypothesis. Another hypothesis that can be advanced in order to explain the GFP over-expression during the chemostat phase involves the characteristics of the GFP variant itself. Indeed, GFPAAV exhibits a C-terminal tag recognized by the ATP-dependent *ClpXP* machinery [21]. The stability of the GFPAAV molecule is thus dependent on the protease content of the cells as well as on ATP availability. These two factors, and particularly the ATP level [28], are known to fluctuate during bioreactor operations, altering the degree of stability of the GFP. The rise in the GFP signal observed during the chemostat phase could thus also be explained by a decrease in the intracellular ATP pool fuelling the *ClpXP* protease complex. Unfortunately, both hypotheses are strengthened by the fact that the GFP signal decreases during the second phase of the chemostat phase (Figure 1, after 48 hours of culture). This observation can be explained either by down-regulation of the *fis* promoter and/or a decrease in the stability of the GFPAAV molecule after the ATP pool is refueled. An explanation about the respective contributions of the different above-mentioned mechanisms could thus be experimentally validated by a complex set of proteomic, transcriptomic and metabolomic profiling of the chemostat phase. In the context of this work, we demonstrate a multiplexed version of the automated FC platform could also be used in order to decipher these dynamics. Prior to that, observations about secondary cytometric variables, i.e. PI uptake by the cells, will be considered in the next section.

### Substrate limitation induces a significant segregation of the population according to PI uptake

**Figure 2:**
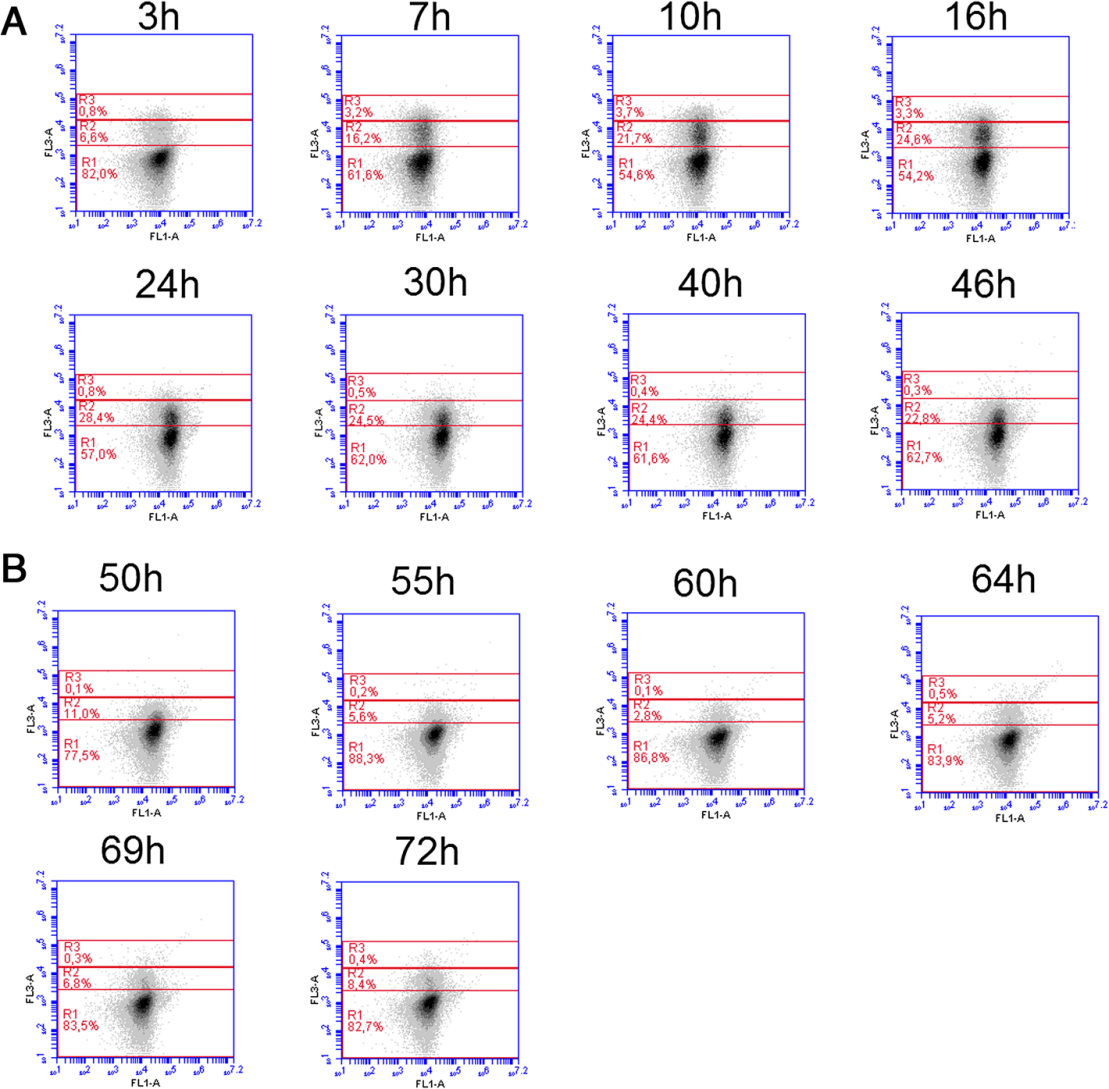
**Evolution of membrane permeability according to PI uptake during A the chemostat phase and B the chemostat phase with additional glucose pulses.** All the results are displayed on a FL3/FL1 dotplot on the basis of the analysis of 40,000 microbial cells by automated flow cytometry. The FL3 channel corresponds to the red fluorescence emitted after PI uptake and the FL1 channels corresponds to the green fluorescence emitted by the GFP synthesized by cells. Representative results from two independent cultivations. *Legend: R1: healthy cells; R2: intermediate cells; R3: damaged cells.*

A significant segregation of the population was noted at the level of the intensity of PI uptake (Figure 2). When the bioreactor shifted from batch mode to chemostat mode, the microbial population was subjected to segregation. Indeed, at the beginning of the culture, no segregation could be observed and the majority of microbial cells were located in the R1 region according to their low PI uptake. However, during the chemostat phase, there was a progressive shift from the R1 region to the R2 region (Figure 2A). When the cells belonging to this R2 subpopulation were sorted, they exhibited the same recovery efficiency as the healthy R1 cells when cultivated on petri dishes (results not shown). The R3 region identified on the FC profiles corresponds to heat inactivated cells (65°C for 30 minutes). This observation is important since the intermediate R2 subpopulation could be responsible for the protein leakage effect noticed at the level of GFP synthesis under the control of the *fis* promoter. In the second phase (Figure 2B), additional glucose pulses were introduced during the chemostat phase. These glucose pulses tended to decrease the segregation between the R1 and R2 subpopulations, with a progressive disappearance of the latter subpopulation. This result is consistent with our previous results [23] as well as with other published works [25,29,30], suggesting that substrate limitation induces an increase in cell membrane permeability in order to increase substrate uptake. The value of the automated FC relies upon its generation of on-line, physiologically related variables that enable direct comparisons of these parameters between each other and with process variables. In our case, FC results suggest that protein leakage could be correlated with the appearance of an R2 subpopulation exhibiting intermediate membrane permeability. However, the intensity of segregation is not taken into account and the use of flow cytograms such as those displayed on Figure 2 is laborious. Accordingly this, the quantification of this segregation will be addressed in a subsequent section. The automated PI staining protocol was further validated by recording several samples during bioreactor operations and comparing them with well-established off-line protocols (Additional file 3: Figure S3) [31].

### Multiplexing potentialities of the automated FC

In order to test the multiplexing potential of our automated FC, additional cultivation tests were carried out on a mini-bioreactors platform. This platform comprised, in our case, three parallel stirred vessels with a working volume of 200 mL each and fully equipped with standard controls. It must be pointed out that, in general, the number of reactors in parallel tends to be increased as much as possible and mini-bioreactor platforms comprising 10 to 24 vessels are common [32,33]. These experiments also allowed exploring the second hypothesis to explain the GFP accumulation under prolonged substrate limitation: a loss of capacity by the cells to degrade the GFP protein. The aim of the experiments was to highlight the equilibrium between GFP caused by *fis* induction, GFP degradation by proteases and the activity of these proteases that are fuelled by ATP.

**Figure 3:**
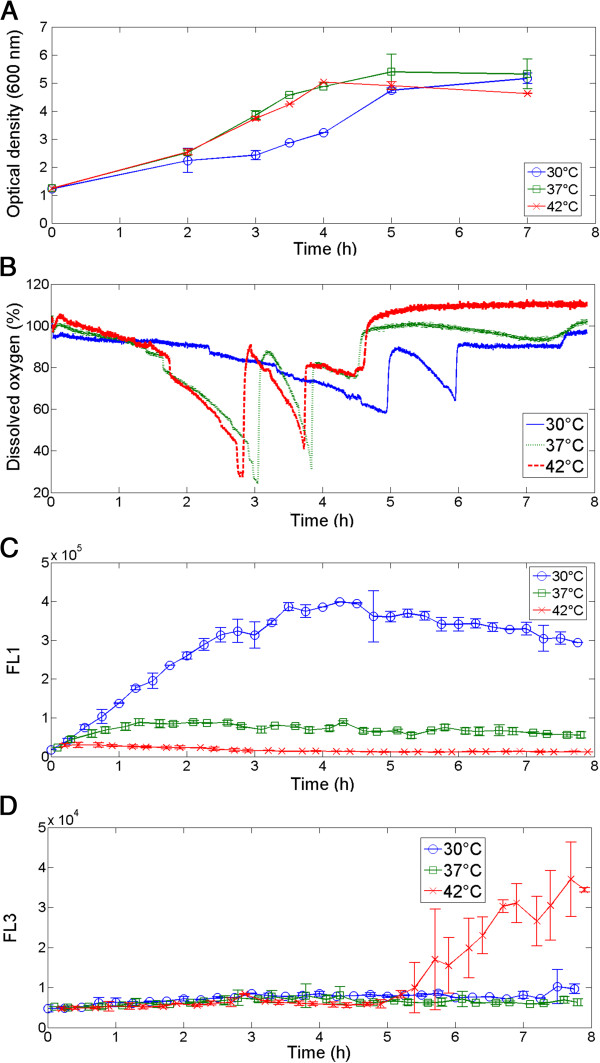
**Follow-up of parallelized cultures of E. *coli pfis::gfpAAV *in 200 mL mini-bioreactors.** The three different cultures have been carried out in batch mode at three temperatures, 30°C, 37°C and 42°C, and monitored by automated flow cytometry. **A**: Evolution of optical density at 600 nm. **B**: Recording of dissolved oxygen profiles. **C**: Monitoring of GFP signal under the control of the *fis* promoter and **D**: PI uptake, by automated flow cytometry. Cultures were performed in duplicate.

In a first test, a simple biological test wa scarried out with three parallel bioreactors running at three different temperatures: 30°C, 37°C end 42°C. At 42°C, the cells exhibited the highest dissolved oxygen consumption (Figure 3B) and the highest growth rate (Figure 3A), while growth was significantly slowed at 30°C (see growth curves and dissolved oxygen consumption Figure 3). Unexpectedly, the GFP signal, which depends on ribosomal activity, decreased when the temperature was increased (Figure 3C). This observation can be explained according to the equilibrium between GFP synthesis, due to *fis* induction, and GFP degradation, linked to ATP-dependent proteases [34,35]. Indeed, a specific carboxyl-terminal oligopeptide extension had been added to render the GFP protein susceptible to degradation, since the normal stability of GFP limits its use for studies of transient gene expression. This tagged protein is recognized and degraded by intracellular tail-specific proteases. It could be supposed that these proteases are more active at 42°C. Indeed, many heat shock proteins are ATP-dependent proteases, and are induced by σ^32^ factor. At 30°C, the level of intracellular σ^32^ is weak, while at 42°C this level increases transiently 15 to 20 fold over 5 min and then stabilizes at a level 2 to 3 fold higher than at 30°C. However, these proteases requires a substantial amount of ATP to be active [36].

**Figure 4:**
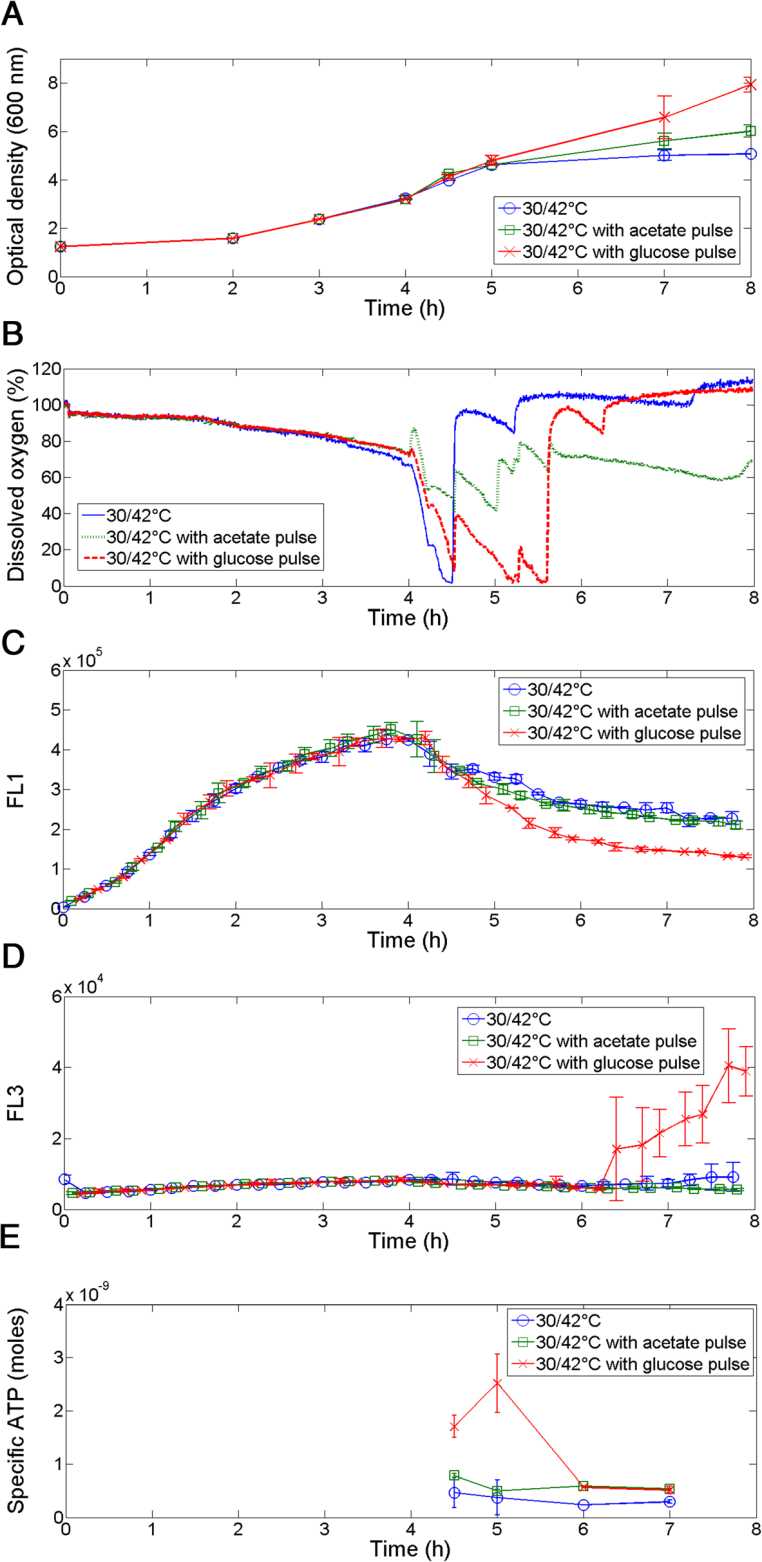
**Follow-up of parallelized cultures of E. *coli pfis::gfpAAV *in 200 mL mini-bioreactors.** The cultures have been initially carried out at 30°C and a sub-lethal heat shock at 42°C has been performed after 4.5 hours of culture. In the second and third mini-bioreactors, acetate and glucose pulse, respectively, were delivered. **A**: Evolution of optical density at 600 nm **B**: Recording of dissolved oxygen profiles. **C**: Monitoring the GFP signal under the control of the *fis* promoter and **D**: PI uptake by automated flow cytometry. **E**: Evolution of specific ATP (ATP content divided by optical density) after 4 h 30 min. Cultures were analysed in duplicate.

**Figure 5:**
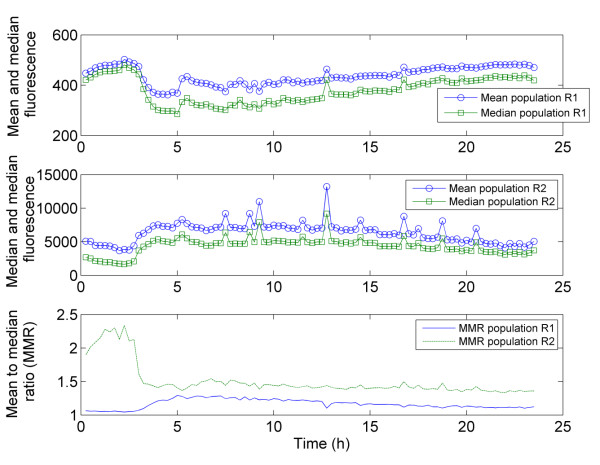
**Evolution of the mean, median and MMR for PI uptake overall and for each subpopulation in the chemostat phase - R1 and R2 - separately.** The gates R1 and R2 corresponds to those displayed on the dotplot of Figure 2.

A second test was then conducted with the three bioreactors in parallel in order to confirm this hypothesis. The three reactors were started at a temperature of 30°C. After 4 h, the temperature was increased to 42°C in order to induce a thermal shock and ATP-dependent protease synthesis. In the second and the third bioreactors, an acetate pulse and a glucose pulse, respectively, were performed at the same time. In these reactors, the consumption of acetate and glucose could be highlighted on-line according to the dissolved oxygen profile. After the substrate pulse, the dissolved oxygen concentration was reduced compared to the reference reactor (no pulse), especially for the case where the glucose pulse had been performed (Figure 4B). In parallel, an obvious increase in growth was noticed in this reactor (Figure 4A). Moreover, in this case, a clear decrease in the GFP signal was noted: indeed, the proteases were massively synthesized at 42°C but required ATP to degrade GFP (Figure 4C). This ATP could be produced via consumption of the added glucose through glycolysis and the Krebs cycle. The ATP concentrations were followed in the three reactors in order to confirm this hypothesis. The ATP levels were significantly higher in the reactor with the glucose pulse, i.e. they increased from 4 h 30 min to 5 h and then decreased as ATP was consumed by proteases degrading the GFP (Figure 5D). Moreover, when ATP was depleted, the membrane permeability increased significantly (Figure 5C). This phenomenon has been previously reported in the literature, where ATP depletion has been correlated with a loss of cellular activity and viability under stress conditions [37,38]. Although these experiments cannot fully explain the complex set of mechanism involved in the activation/deactivation of the *pfis::gfpAAV* bio-reporter, multiplexed FC demonstrated a clear correlation between GFP stability and ATP availability, suggesting that transcriptional control of the *fis* promoter is not the only mechanism involved.

This set of experiments demonstrated the value of using a simple flow cytometer interface that can be multiplexed with several bioreactors in order to perform high throughput experiments and gain insights at the level of complex biological processes under dynamic conditions.

### Towards a definition of variables for on-line characterization of population segregation in bioreactors

Since phenotypic heterogeneity is known to occur during bioprocesses, the use of FC as a single-cell sensor is a very promising alternative that has been considered only in a limited amount of studies. Indeed, automated FC is still rarely used for bioprocess optimization considering the difficulty in properly interfacing with the process equipment. On the other hand, significant efforts have been made toward the automated analysis of FC data and, more particularly, for the description of sub-populations by appropriate gating. However, these algorithms are not actually embedded in standard FC software and the analysis must be performed a posteriori [39]. From the perspective of an application for bioreactor control this is an issue since the FC data have to be analysed on-line in order to enable feedback regulation. As a first case study, PI uptake dynamics were followed more in detail during the transition from the batch phase to the chemostat phase, considering that a significant segregation of the microbial population according to PI uptake has been observed during this transition. Segregation can be determined on the basis of the respective percentages belonging to two (or more) subpopulations. From a mathematical perspective, it is possible to define a degree of segregation varying from 0 (minimal segregation when 100% of the cells are located in a single gate) to 1 (maximal segregation when the cells are equally partitioned between the two subpopulations) according to the relative percentage of cells between the two states:

Degree of segregation=1-|%R1-%R2|

where %R1 is the percentage of PI negative cells and %R2 the percentage of PI intermediate cells, whereas || stands for the absolute value of the difference between the two percentages.

**Figure 6:**
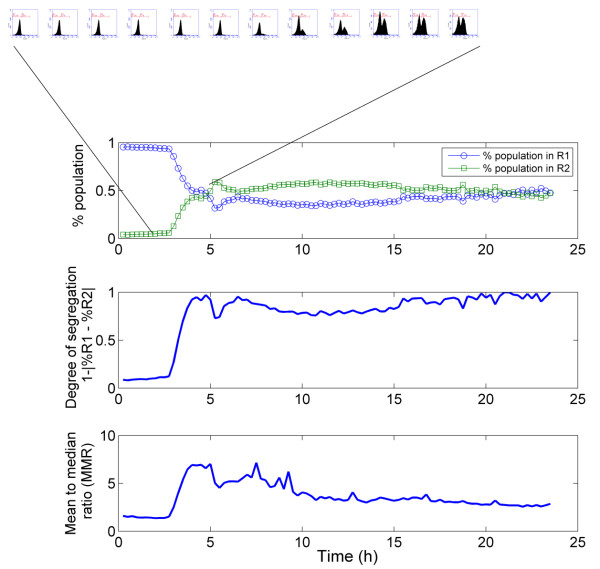
**Characterization of the evolution of the segregation phenomenon during the transition from batch to chemostat mode (the transition occurs after 3 hours; for more information about the culture, refer to Figures**1** and **2**) using different approaches, i.e. from top to bottom, evolution in the percentage of cells in the PI negative (population in gate R1) and PI positive (population in gate R2) state, the degree of segregation computed from the above-mentioned percentage and the evolution of the mean to median ratio (MMR).** Some histograms depicting the distribution of PI among the population are displayed in order to show the segregation dynamics.

If we apply this principle to the data (Figure 6), maximum segregation is reached after 4 hours, in accordance with the transition from the batch culture to the chemostat mode and with the evolution of the respective percentages in R1 and R2. However, this technique cannot be applied to the on-line treatment of FC data since it requires gating the results. Another way to analyse the degree of the segregation is to look simultaneously at different values expressing the central tendency of the distribution. In our case, the mean and median of the cytometric variables are automatically computed at the level of the embedded software. Both approaches were applied to the set of chemostat data and showed some differences after 15 h (Figure 6). Indeed, after a sharp increase following 3 hours of cultivation, the MMR tends to decrease continuously for the remainder of the experiment, whereas the degree of segregation increases again after 15 hours of culture according to a redistribution of the cells between R1 and R2. However, the segregation phenomenon depends not only on the numbers of cells in the respective sub-populations, but also on the values of the cytometric variables within the sub-population. In this context it must be noticed that PI intensity exhibits a decrease after the initial establishment of segregation at the level of the culture (Figure 2A). In this respect the MMR seems to be a better estimate of segregation, integrating both the relative numbers of cells among each subpopulation as well as the distance between the values of the cytometric variables in the two sub-populations. In order to assess the efficiency of the MMR as a good indicator of microbial population segregation, it is important to look at the evolution of this ratio when no segregation phenomenon is observed. The MMR was thus computed for each subpopulation R1 and R2 separately (Figure 5). The higher value for the MMR noted for the subpopulation in R2 at the beginning of the culture can be attributed to the fact that only a very few cells are present in this state (less than 5% of the cells at the beginning of the culture), altering the relevance of the mean and the median. However, when both regions contains a sufficient number of individuals, the MMR is close to 1 for each subpopulation and remains so for the duration of the culture, suggesting that higher MMR values are only noted in cases of significant segregation of the population.

**Figure 7:**
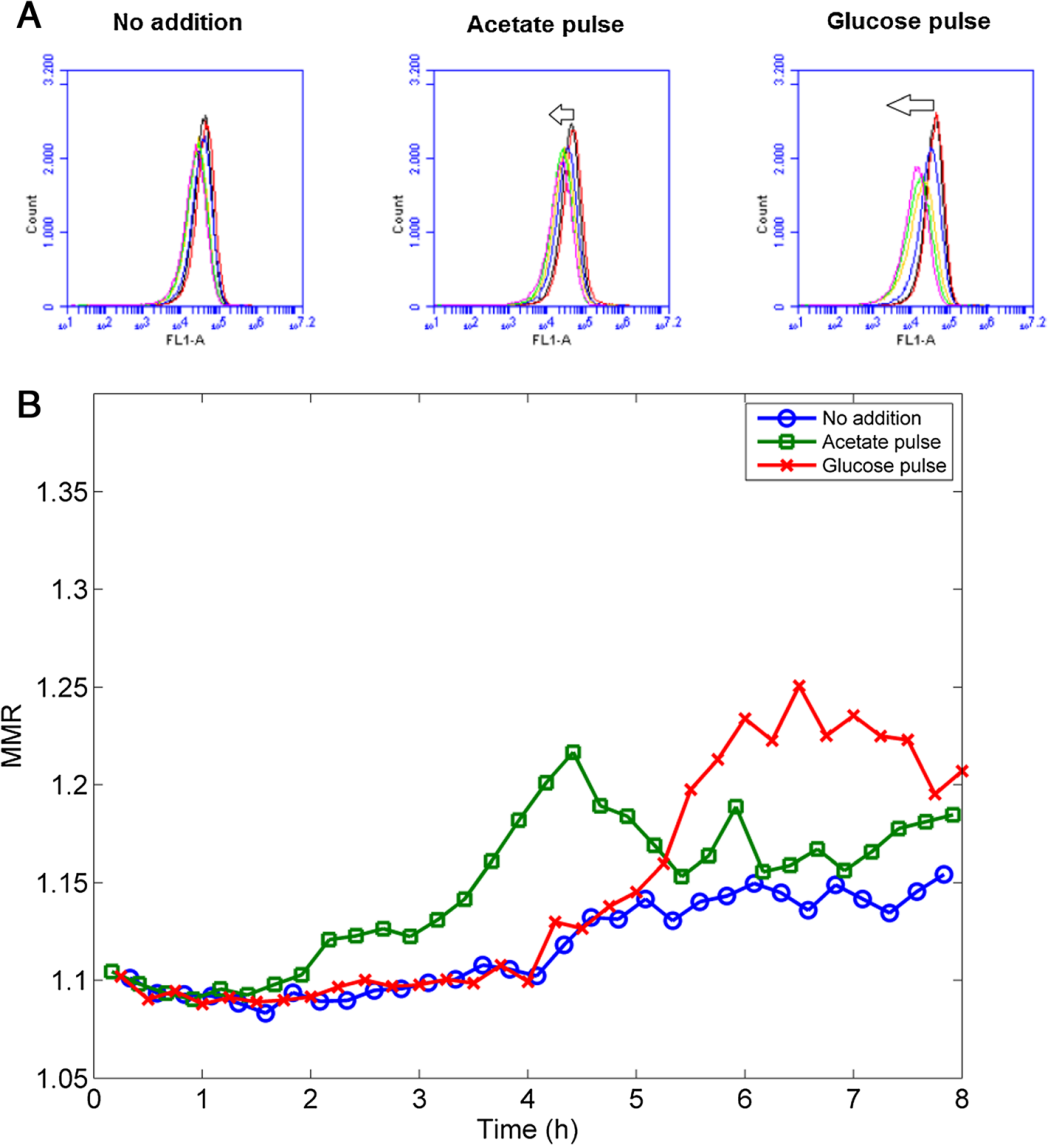
**Estimation of the segregation intensity during multiplexed experiments. A**: Evolution of the GFP distribution for the parallel culture carried out after heat shock (the arrow indicates the progression of the distribution from the initial heat shock to the end of the culture; for more details about the kinetics, please refer to Figure 4). **B**: Evolution of the MMR for the three parallel bioreactors carried out with heat shock.

The validity of the MMR was also assessed in the case of the multiplexed bioreactor experiments at the level of single-cell GFP content after the application of heat shock, where no segregation was observed but an increase in the standard deviation was noted (Figure 7). When a glucose pulse was added after the heat shock, a significant decrease in GFP intensity was reported, considered to reflect degradation by ATP-dependent proteases (Figure 4C). This decrease was also noticed at the level of the GFP histogram, in which the distribution became wider when protein degradation occurred (Figure 7A). This phenomenon was noticed to a lesser extent when an acetate pulse was delivered, and almost no change in distribution was observed when no substrate was added. The MMR was calculated for each bioreactor (Figure 7B) and its value stayed close to 1 under each condition, suggesting anew that the MMR is only significantly affected when segregation occurs. This parameter can be thus used directly on the raw data and is independent from any gating operations which are user specific and leads to variability at the level of the interpretation of FC data [40].

## Conclusion

A low-cost FC platform has been designed in order to follow both GFP synthesis and PI uptake inside bioreactors. This system is simple, robust and gives reliable results compared with off-line analysis. However, one of the main limitations of FC is its requirement for fluorescent tag for physiologically relevant analysis. In the context of this work, a *pfis::gfpAAV* bio-reporter was used in order to track nutrient status at the single cell level. Automation of FC analysis is particularly useful in the case of a destabilized reporter, since off-line analysis requires sample processing that can affect the quality of the results. However, our results have shown that the response of the *pfis::gfpAAV* system is altered by unknown physiological mechanisms attributable either to up-regulation of the *fis* promoter or to the destabilized GFP itself. In this context, a multiplexed version of automated FC allowed us to demonstrate that the degree of stabilization of the GFPAAV is involved in this process and is correlated to the intracellular ATP content of the cells. Finally, MMR has been proposed as a useful parameter for on-line detection of microbial population segregation. Overall, this work demonstrates that a simplified version of on-line FC can be used at the process level or in a multiplexed version to investigate the dynamics of complex physiological mechanisms. In this respect, the determination of new on-line parameters (e.g. the MMR) is of primary importance in order to fully integrate the power of FC into dedicated feedback control loops.

## Methods

### Microbial GFP reporter strain and medium

*Escherichia coli* K12 MG1655 bearing a pGS20 plasmid with a *pfis::gfpAAV* gene and a chloramphenicol resistance gene was used in this work. Additional details about strain construction can be found in [18]. pGS20PfisBAAV was digested with NotI and NdeI to replace the rrnB promoter region with the E. coli *fis* promoter that encodes a DNA binding protein, FIS. The *fis* promoter was amplified from the *E. coli* chromosome using primers FisP-SD (this primer contains an optimized SD sequence) and FisP-up. The 300 bp PCR product was subsequently digested with NotI and NdeI and ligated into the vector fragment. The resulting plasmid was called pGS20FisGFPAAV (10–15 copies/cell, chloramphenicol resistant marker, 25 μg/mL) [18].

The strain was maintained at -80°C in working seed vials (2 ml). Pre-cultures and cultures were grown on a defined mineral salt medium containing (in g/L): K2HPO4 14.6, NaH2PO4.2H2O 3.6, Na2SO4 2.0, (NH4)2SO4 2.47, NH4Cl 0.5, (NH4)2-H-citrate 1.0, glucose 5.0, thiamine 0.01, and chloramphenicol 0.05. Thiamine and kanamycin were sterilized by filtration (0.2 μm). The medium was supplemented with 3 mL/L trace solution, 3 mL/L FeCl3.6H2O solution (16.7 g/L), 3 mL/L EDTA solution (20.1 g/L) and 2 mL/L MgSO4 solution (120 g/L). The trace solution contained (in g/L): CoCl2.H2O 0.74, ZnSO4.7H2O 0.18, MnSO4.H2O 0.1, CuSO4.5H2O 0.1, and CoSO4.7H_2_O 0.21. Before operating in the bioreactor, a pre-cultivation step was performed in 100 mL of the above-mentioned medium in a baffled shaker flask at 37°C under orbital shaking at 140 rounds per minute during 16 h.

### Automated FC protocol

**Figure 8:**
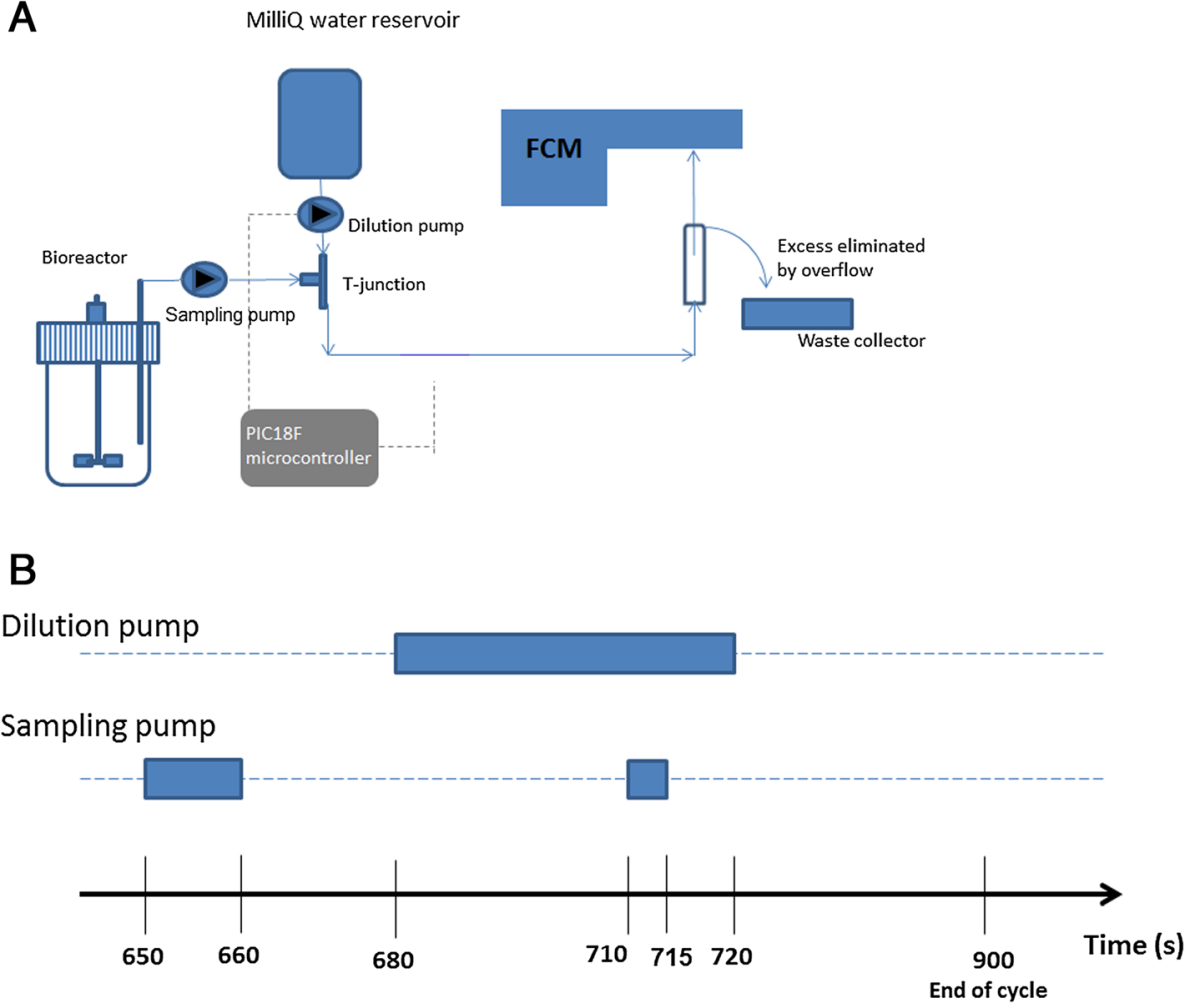
**Automated FC set-up. A**: Scheme of the interfacing system between the bioreactor and the FC; **B**: Sequence of activation of the dilution and sampling pump. First, activation of the sampling pump to fill the line and, second, activation simultaneously with the dilution pump to reach an appropriate dilution of the sample before FC analysis.

The interfacing system between the bioreactor (see the next section for a detailed description of bioreactor set up and operating mode) and BD Accuri C6 FC was constructed on the basis of the in-line mixing principle (Figure 8). Detailed informations about the Accuri C6 FC can be found online [41]. A first peristaltic pump (sampling pump, Watson Marlow 040.DP1D.N2R operating at a rate of 32 mL/min) extracted a cell suspension sample directly from the bioreactor and injected it in a diluting flow of water (milliQ water as the sheat fluid of the C6 FC). The dilution fluid was carried forward by a second peristaltic pump (dilution pump, Watson Marlow 040.DP1D.N2R operating at a rate of 32 mL/min). In line mixing was achieved by a polypropylene T-mixer. Silicon tubing (internal diameter: 1 mm) was used except for the direct insertion into the peristaltic pump, where marprene was used in order to increase the shelf life of the tubing. All the connections were made using male polypropylene luer lock systems (internal diameter: 1.6 mm). The sequence of activation of the peristaltic pumps was controlled automatically by a PIC18F microcontroller. The PIC microcontroller was embedded in a full PICPLC16 control card provided by Mikroelektronika. This card comprised several relays that were programmed in order to activate or deactivate the peristaltic pumps. The control procedure was designed on the basis of a C code written using the MikroC pro for PIC controller software (the different sequences are illustrated in Figure 8 and the code is provided in Additional file 4). These sequences of activation/deactivation were set up on the basis of two constraints. The first constraint involved full cleaning of the sampling lines in order to avoid cross-contamination between successive samples. The second constraint implies an appropriate dilution of the cell suspension before FC analysis. The in-line mixing system was adjusted so that samples for flow cytometry analysis always comprised less than 3.10^6^ cells/mL in order to be in the linear range of analysis and to achieve an effective separation of the events (Additional file 5: Figure S4). The sampling sequence was repeated each 15 minutes. For each sequence, the FC analysis was activated automatically. The sample was pumped through the FC at a flow rate of 33 μL/min and the resulting events were recorded for 1 minute and limited to 40,000 events. For each event, the mean and median FL1 parameter (green fluorescence related to GFP synthesis) and the mean and median FL3 parameter (red fluorescence related to propidium iodide uptake) were recorded. Propidium iodide staining was performed by adding PI directly into the milliQ reservoir at a concentration of 10 mg/L. Both the contact time and the concentration of PI solution are recognized as critical parameters for appropriate staining of environmental bacteria [42]. However, in this work a well-known industrial strain of *E. coli* was used and a large number of PI staining protocols are available in the literature for this strain. According to these protocols, the contact time can vary from 10–15 minutes [43] to 20–25 minutes [31]. For this work, we determined the minimum contact time between cells and PI necessary in order to optimize the rate of data acquisition by the automated FC. The control procedure involved an off-line PI staining test involving different contact times and a real-time FC test. In this way a minimum contact time of 3 minutes was determined to be necessary, and was incorporated in the automated FC procedure (Additional file 6: Figure S5). Data treatment was performed by a dedicated Matlab code after export from the CFlow software as a .csv file. PI stained cells were sorted (FACSaria, BectonDickinson) and plated onto agar medium (glucose 10 g/L, yeast extract 10 g/L, casein peptone 10 g/L, agar 15 g/L) in order to assess their viability. GFP and PI measures were compared to those obtained off-line (Additional file 1: Figure S1 and Additional file 3: Figure S3). For off-line staining tests, cells were stained with iodide propidium (PI, Sigma Aldrich Fluka, Saint-Louis Missouri USA), which accumulates in membrane-compromised cells. A quantity of 10 μl of PI (working solution 1 mg/mL of PI in distilled water) was used during 15 min at 37°C. PI was measured on the FL3 channel of the C6Flow Accuri Cytometer (BD Biosciences, NJ USA). The analysis was stopped after the acquisition of 30,000 events, with a threshold of 80,000 set on the FSC channel. Flow cytograms of FL3/FL1 were analysed by CFlowPlus Analysis Software. The GFP signal under the control of the *fis* promoter was recorded at the level of the FL1 channel (excitation wavelength: 488 nm; emission wavelength: 530 nm). The cells were stored at - 20°C in glycerol (30%). These stabilized cells were centrifuged and resuspended in PBS buffer before analysis.

### Bioreactor operating conditions

Chemostats were carried out in a lab-scale stirred bioreactor (Biostat B-Twin, Sartorius with a total volume of 3 L; working volume of 1 L; mixing provided by a standard Rushton disk turbine with 6 blades) in remote control mode interfaced with the MFCS/win 3.0 software. During the experiments, pH was maintained at 6.9 (regulation by ammonia and phosphoric acid) the stirrer rate at 800 rpm, the air flow rate at 1 L/min and the temperature at 37°C. The chemostat phase was stabilized during 43 h with a feeding solution containing 5 g/L of glucose solution (in the same minimal medium as previously described) at a dilution rate of 0.14 h^-1^(corresponding to 6 residence times). Glucose pulses were performed during 24 h at the same dilution rate by adding pulses of 8 mL of a glucose solution (30 g/L made up in distilled water) each 15 minutes. Additional batch experiments were performed on a Dasgip mini-bioreactor platform. The mini-bioreactors were filled with 200 mL of the defined medium previously described. Stirring was provided by two Rushton turbines with 6 blades at an agitation rate of 900 min-1. The air flow was maintained at 100 mL/min and the pH was kept at 7.0. The first experiments involved three bioreactors at three different temperatures: 30°C, 37°C and 42°C. A second experiment was also performed. The three reactors were started at 30°C. After 4 h, the temperature was increased in the reactors to 42°C to induce a thermal shock and ATP-dependent protease synthesis. In the second and the third bioreactors, an acetate pulse and a glucose pulse, respectively, were delivered at the same time (2 g added in total). All these experiments were carried out with two replicates. At both scales, cell growth was monitored by optical density (OD) at a wavelength of 600 nm (Genesys 105 UV–VIS spectrophotometer, purchased from Thermo Scientific). Cell dry weight was determined on the basis of filtered samples (0.45 μm) dried during 24 h at 105°C.

Glucose concentration was monitored by a YSI (Yellow Spring Instrument Co) electro-enzymatic system. Samples were injected into a chamber filled with a buffer solution and the sample was diffused through a polycarbonate membrane that limited the reaction rate. Membrane-immobilised glucose oxidase produced hydrogen peroxide that diffused through a cellulose acetate membrane and was oxidized by a palatine electrode. The signal was proportional to the glucose concentration up to 2.5 g/L. More concentrated samples were diluted first. The total amount of protein in the extracellular medium was measured on the basis of the Folin-Lowry method [44].

ATP assays were performed on supernatants from the mini-bioreactors according to the CellTiteGlo® luminescent assay. The assay procedure involved adding a simple reagent directly to the cells. This resulted in cell lysis and generation of a luminescent signal proportional to the amount of ATP present, measured by a V^3^ Wallac luminometer (Perkin Elmer). This relies on the properties of a thermostable luciferase that generates a stable luminescent signal for 5 hours: mono-oxygenation of luciferin is catalysed by luciferase in the presence of Mg2^+^, ATP and molecular oxygen. One hundred microlitres of reagent was added to 100 μl of culture medium (the cell concentration has to be less than 5.10^4^ cell/ml). A calibration curve was done over the same measurement range. All analyses were performed in triplicates and expressed as specific ATP, i.e. the ATP concentration divided by cell optical density.

## Competing interests

The authors declare that they have no competing interests.

## Authors’ contributions

AB and FD performed the cultivation tests and flow cytometry analyses. FD and FL designed the interfacing of the flow cytometer. AB and FD wrote the paper. SSH and SS constructed the bacterial strain and helped in editing and revising the manuscript. FD and PT supervised the work and corrected the paper. All authors read and approved the final version of the manuscript.

## Supplementary Material

Additional file 1: Figure S1 Comparison of the on-line and off-line FL1 values (GFP synthesis). Click here for file

Additional file 2: Figure S2 Evolution of the extracellular protein concentration in 2 L bioreactors. Click here for file

Additional file 3: Figure S3. Comparison of the on-line and off-line FL3 values (PI uptake). Click here for file

Additional file 4 C codes written for the PIC controller corresponding to the chemostat culture and the multiplexed version for mini-bioreactors. Click here for file

Additional file 5: Figure S4 Determination of the linear range of analysis of the accuri C6 flow cytometer. Click here for file

Additional file 6: Figure S5 Real-time monitoring of PI uptake and comparison with off-line staining. Click here for file
