# Supplementary material for: A low-cost, multiplexable, automated flow cytometry procedure for the characterization of microbial stress dynamics in bioreactors
Source: Microb Cell Fact. 2013 Oct 31;12:100. doi: 10.1186/1475-2859-12-100 (PMC4228430; doi:10.1186/1475-2859-12-100)
Supplement: Additional file 1: Figure S1 — Comparison of the on-line and off-line FL1 values (GFP synthesis). [file 1475-2859-12-100-S1.doc]

**Supplementary file S1**

**Comparison of the on-line and off-line FL1 values (GFP synthesis)**

By using the dilution protocol displayed in supplementary file S1 and described in the material and methods section, no significant difference can be observed between the samples acquired manually and by automated FC. When no dilution is performed, the GFP signal recorded by automated FC is higher accounting for an ineffective separation of the microbial cells at the level of the automated FC

**
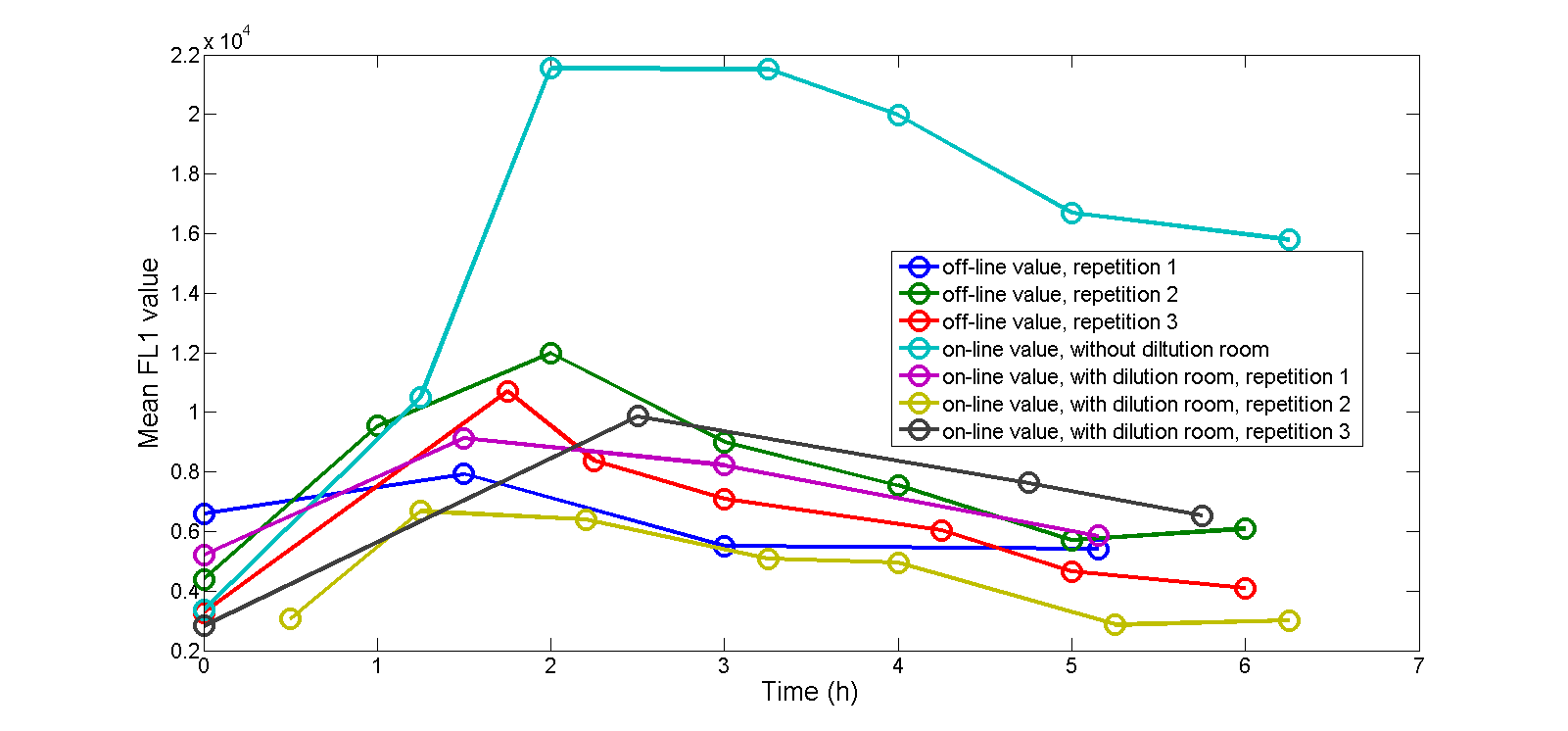
**
