# Supplementary material for: A low-cost, multiplexable, automated flow cytometry procedure for the characterization of microbial stress dynamics in bioreactors
Source: Microb Cell Fact. 2013 Oct 31;12:100. doi: 10.1186/1475-2859-12-100 (PMC4228430; doi:10.1186/1475-2859-12-100)

**Supplementary file S2**

**Evolution of the extracellular protein concentration in 2L bioreactors**

**Figure S2 : evolution of the extracellular protein concentration in 2L bioreactors**

Evolution of the extracellular proteins and amino acids concentration in function of the time during the chemostat culture without and with glucose oscillations in 2l bioreactor. Protein leakage can be mainly observed during the chemostat phase D = 0.14 h^-1^ with substrate limitation. These proteins are subsequently reconsumed during the second phase with glucose oscillations (after 48 h).


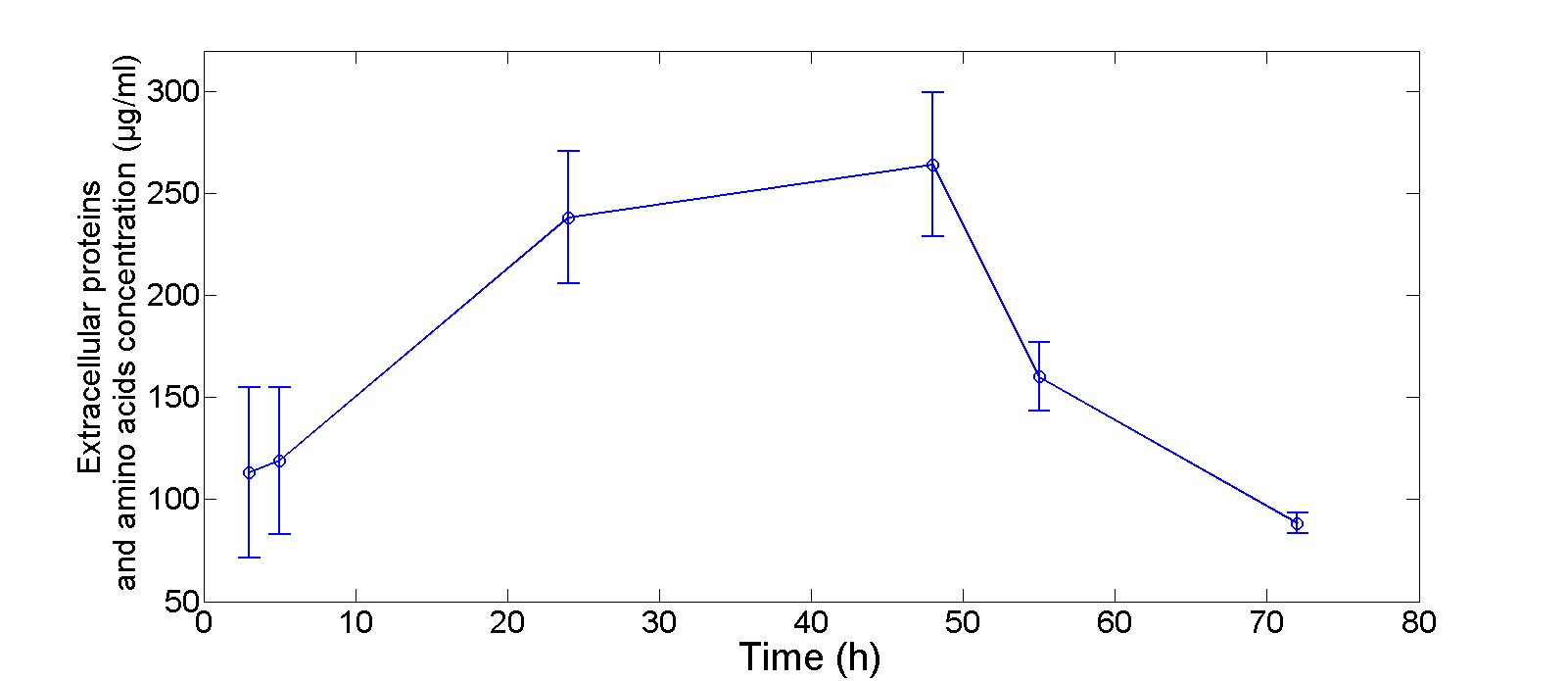

Supplement: Additional file 2: Figure S2 — Evolution of the extracellular protein concentration in 2 L bioreactors. [file 1475-2859-12-100-S2.doc]
