# Supplementary material for: A low-cost, multiplexable, automated flow cytometry procedure for the characterization of microbial stress dynamics in bioreactors
Source: Microb Cell Fact. 2013 Oct 31;12:100. doi: 10.1186/1475-2859-12-100 (PMC4228430; doi:10.1186/1475-2859-12-100)

**Supplementary file S3**

**Comparison of the on-line and off-line FL3 values (PI uptake)**

Comparison of on-line and off-line flow cytograms FL3 (PI uptake) and FL1 (GFP synthesis). Off-line samples have been stained with 10 µL of PI solution (1 mg/mL) for 15 minutes before FC analysis. Representative results of two independent cultivations.


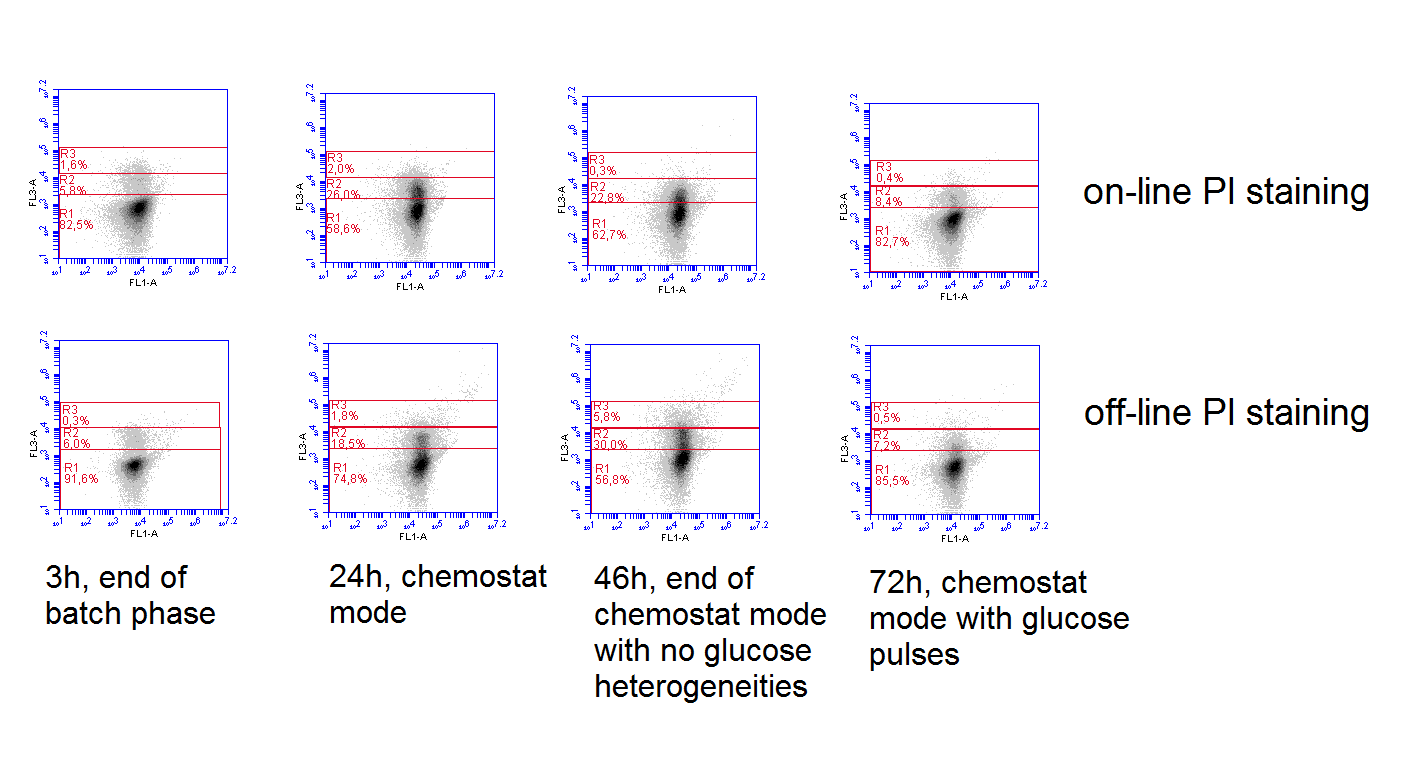

Supplement: Additional file 3: Figure S3. — Comparison of the on-line and off-line FL3 values (PI uptake). [file 1475-2859-12-100-S3.doc]
