# Supplementary material for: A low-cost, multiplexable, automated flow cytometry procedure for the characterization of microbial stress dynamics in bioreactors
Source: Microb Cell Fact. 2013 Oct 31;12:100. doi: 10.1186/1475-2859-12-100 (PMC4228430; doi:10.1186/1475-2859-12-100)
Supplement: Additional file 4 — C codes written for the PIC controller corresponding to the chemostat culture and the multiplexed version for mini-bioreactors. [file 1475-2859-12-100-S4.doc]

**Supplementary file S6**

C codes written for the PIC controller corresponding to the chemostat culture and the multiplexed version for mini-bioreactors

- *Chemostat culture*

// PIC18F4520 quartz 8 MHz PLL NON

// MIKROC Pro 5.40

//--------------------- --------------------------------------------------------

#define LFEED UART1_Write_Text("\n\r")

#define POMPE_EAU LATB.B0 // RLY1

#define POMPE_FERMENTEUR LATB.B1 // RLY2

#define POMPE_FERMENTEUR2 LATB.B2 // RLY3

#define POMPE_GLUCOSE LATB.B3 // RLY4

#define NIVEAU_haut 600 //

#define NIVEAU_bas 200 //

//------------------------------------------------------------------------------

unsigned char timer0_flag=0; //

unsigned char GO_flag=1;

unsigned char UNLOCK_flag=0;

unsigned char MODE_ANALYSE=0; //

unsigned int chrono_sec=0; //

unsigned int chrono_synchro=0; //

unsigned int TENSION=0; //

char txtint[7]; //

int DELTA_T=0; //

unsigned int TOP_FIN=0; //

//------------------------------------------------------------------------------

void mesure_TENSION(){ //

char z;

unsigned int tmp;

for(z=0;z<4;z++){ //

tmp=tmp + ADC_Read(0);

}

TENSION = tmp>>2 ; //

}

//------------------------------------------------------------------------------

//------------------------------------------------------------------------------

void interrupt() {

if (TMR0IF_bit) { // overflow TIMER0

timer0_flag=1;

chrono_sec++;

//if (UNLOCK_flag==1) chrono_synchro++;

TMR0H = 0xC2; TMR0L = 0xF7; // TIMER0 chargé avec 49911

TMR0IF_bit = 0; // clear TMR0IF

}// end if TMROIF_bit

}// end if interrupt

//------------------------------------------------------------------------------

void main() {

UART1_Init(9600);

delay_ms(100);

UART1_Write_Text("FERMENTEUR v1.0 11-07-2012");LFEED;

CVRCON.B7=0; //

CVRCON.B6=0; //

CMCON=0b00000000; //

ADCON0.B0=1; // ADC module ON

TRISA.B0=1; // ADCON1=0b1110; //

TRISB.B0 = 0; // RB0 en output RLY1 POMPE_EAU

TRISB.B1 = 0; // RB1 en output RLY2 POMPE_FERMENTEUR

TRISB.B2 = 0; // RB2 en output RLY3 POMPE_FERMENTEUR2

TRISB.B3 = 0; // RB3 en output RLY4 POMPE_GLUCOSE

INTCON = 0xE0; // autorise interrupt timer0

T08BIT_bit =0; //

T0CS_bit =0; //

PSA_bit =0; //

T0PS2_bit= 1 ; T0PS1_bit= 1 ; T0PS0_bit= 0 ;

// prescaler 110 = division par 128

// quartz de 8 MHz ==>> CLK =OSC/4 ===> 2 MHz

// 2 MHz / 128 ==>

TMR0L= 0xF7; //

TMR0H= 0xC2; //

POMPE_EAU = 0; //

POMPE_FERMENTEUR=0;

POMPE_FERMENTEUR2=0;

POMPE_GLUCOSE=0;

MODE_ANALYSE=0;

while(1){

if(timer0_flag==1){ // +/- 1 Hz

mesure_TENSION();

if(POMPE_EAU==1 && TENSION > NIVEAU_haut && MODE_ANALYSE==0 ){ MODE_ANALYSE=1; }

if(POMPE_EAU==1 && TENSION < NIVEAU_bas && MODE_ANALYSE==1 ){ //

MODE_ANALYSE=0;

UNLOCK_flag=1; }

//infos debug

IntToStr(TENSION, txtint);

UART1_Write_Text("AN0 : ");UART1_Write_Text(txtint);LFEED;

IntToStr(GO_flag, txtint);

UART1_Write_Text("GO_f: ");UART1_Write_Text(txtint);LFEED;

IntToStr(chrono_sec, txtint);

UART1_Write_Text("chse: ");UART1_Write_Text(txtint);LFEED;

IntToStr(MODE_ANALYSE, txtint);

UART1_Write_Text("MOAn: ");UART1_Write_Text(txtint);LFEED;

IntToStr(chrono_synchro, txtint);

UART1_Write_Text("chsy: ");UART1_Write_Text(txtint);LFEED;

IntToStr(UNLOCK_flag, txtint);

UART1_Write_Text("UnlF: ");UART1_Write_Text(txtint);LFEED;LFEED;

//fin infos debug

timer0_flag=0;

}

if(GO_flag==1){

if(chrono_sec==1){

POMPE_GLUCOSE=1;

}

if(chrono_sec==21) {

POMPE_GLUCOSE=0;

}

if(chrono_sec==650){

POMPE_FERMENTEUR=1; //1

}

if(chrono_sec==660){

POMPE_FERMENTEUR=0;

}

if(chrono_sec==680){ //680

POMPE_EAU=1; //

}

if(chrono_sec==710){ //700

POMPE_FERMENTEUR=1; //1

}

if(chrono_sec==715){ //702 705

POMPE_FERMENTEUR=0; //0

}

if(chrono_sec==720){ //715 DTS : 60

POMPE_EAU=0; // DTS

GO_flag=0;

}

}// end go flag =1

if( GO_flag==0 && chrono_sec>900){

GO_flag=1;

chrono_sec=0;

chrono_synchro=0;

}

}// end while

}//end main

//------------------------------------------------------------------------------

- *Mini-bioreactors*

// PIC18F4520 quartz 8 MHz PLL NON

// MIKROC Pro 5.40

//--------------------- --------------------------------------------------------

#define LFEED UART1_Write_Text("\n\r")

#define POMPE_EAU LATB.B0 // RLY1

#define POMPE_FERMENTEUR LATB.B1 // RLY2

#define POMPE_FERMENTEUR2 LATB.B2 // RLY3

#define POMPE_FERMENTEUR3 LATB.B3 // RLY4

#define POMPE_GLUCOSE2 LATB.B4 // RLY5

#define POMPE_GLUCOSE3 LATB.B5 // RLY6

#define NIVEAU_haut 600 //

#define NIVEAU_bas 200 //

//------------------------------------------------------------------------------

unsigned char timer0_flag=0; //

unsigned char GO_flag=1;

unsigned char UNLOCK_flag=0;

unsigned char MODE_ANALYSE=0; //

unsigned int chrono_sec=0; //

unsigned int chrono_synchro=0; //

unsigned int TENSION=0; //

char txtint[7]; //

int DELTA_T=0; //

unsigned int TOP_FIN=0; //

//------------------------------------------------------------------------------

void mesure_TENSION(){ //

char z;

unsigned int tmp;

for(z=0;z<4;z++){ //

tmp=tmp + ADC_Read(0);

}

TENSION = tmp>>2 ; //

}

//------------------------------------------------------------------------------

//------------------------------------------------------------------------------

void interrupt() {

if (TMR0IF_bit) { //

timer0_flag=1;

chrono_sec++;

//if (UNLOCK_flag==1) chrono_synchro++;

TMR0H = 0xC2; TMR0L = 0xF7; //

TMR0IF_bit = 0; //

}// end if TMROIF_bit

}// end if interrupt

//------------------------------------------------------------------------------

void main() {

UART1_Init(9600);

delay_ms(100);

UART1_Write_Text("FERMENTEUR v1.0 11-07-2012");LFEED;

CVRCON.B7=0; // CVRCON.B6=0; //

CMCON=0b00000000; //

ADCON0.B0=1; //

TRISA.B0=1; //

ADCON1=0b1110; //

TRISB.B0 = 0; // RB0 en output RLY1 POMPE_EAU

TRISB.B1 = 0; // RB1 en output RLY2 POMPE_FERMENTEUR

TRISB.B2 = 0; // RB2 en output RLY3 POMPE_FERMENTEUR2

TRISB.B3 = 0; // RB3 en output RLY4 POMPE_FERMENTEUR3

TRISB.B4 = 0; // RB4 en output RLY5 POMPE_GLUCOSE2

TRISB.B5 = 0; // RB5 en output RLY5 POMPE_GLUCOSE3

INTCON = 0xE0; //

T08BIT_bit =0; //

T0CS_bit =0; //

PSA_bit =0; //

T0PS2_bit= 1 ; T0PS1_bit= 1 ; T0PS0_bit= 0 ;

// prescaler 110 = division par 128

// quartz de 8 MHz ==>> CLK =OSC/4 ===> 2 MHz

// 2 MHz / 128 ==> TMR0 est incrémenté 15625 fois par seconde

// un compteur de 16 bits ( 0 à 65535 ) déborde en 4.194 secondes

TMR0L= 0xF7; //

TMR0H= 0xC2; //

POMPE_EAU = 0; //

POMPE_FERMENTEUR=0;

POMPE_FERMENTEUR3=0;

POMPE_FERMENTEUR2=0;

POMPE_GLUCOSE2=0;

POMPE_GLUCOSE3=0;

MODE_ANALYSE=0;

while(1){

if(timer0_flag==1){ // +/- 1 Hz

mesure_TENSION();

if(POMPE_EAU==1 && TENSION > NIVEAU_haut && MODE_ANALYSE==0 ){ //

MODE_ANALYSE=1;

}

if(POMPE_EAU==1 && TENSION < NIVEAU_bas && MODE_ANALYSE==1 ){ //

MODE_ANALYSE=0;

UNLOCK_flag=1; //

}

//infos debug

IntToStr(TENSION, txtint);

UART1_Write_Text("AN0 : ");UART1_Write_Text(txtint);LFEED;

IntToStr(GO_flag, txtint);

UART1_Write_Text("GO_f: ");UART1_Write_Text(txtint);LFEED;

IntToStr(chrono_sec, txtint);

UART1_Write_Text("chse: ");UART1_Write_Text(txtint);LFEED;

IntToStr(MODE_ANALYSE, txtint);

UART1_Write_Text("MOAn: ");UART1_Write_Text(txtint);LFEED;

IntToStr(chrono_synchro, txtint);

UART1_Write_Text("chsy: ");UART1_Write_Text(txtint);LFEED;

IntToStr(UNLOCK_flag, txtint);

UART1_Write_Text("UnlF: ");UART1_Write_Text(txtint);LFEED;LFEED;

//fin infos debug

timer0_flag=0;

}

if(GO_flag==1){

if(chrono_sec==350-300){ //

POMPE_FERMENTEUR=1; //

}

if(chrono_sec==365-300){ //

POMPE_FERMENTEUR=0;

}

if(chrono_sec==380-300){ //

POMPE_EAU=1; //

}

if(chrono_sec==410-300){ //

POMPE_FERMENTEUR=1; //

}

if(chrono_sec==415-300){ //

POMPE_FERMENTEUR=0; //

}

if(chrono_sec==420-300){ //

POMPE_EAU=0; //

}

//FERMENTEUR 2

if(chrono_sec==950-600){ //

POMPE_FERMENTEUR2=1; //

}

if(chrono_sec==965-600){ //

POMPE_FERMENTEUR2=0;

}

if(chrono_sec==980-600){ //

POMPE_EAU=1; //

}

if(chrono_sec==1010-600){ //

POMPE_FERMENTEUR2=1; //

}

if(chrono_sec==1015-600){ //

POMPE_FERMENTEUR2=0; //

}

if(chrono_sec==1020-600){ //

POMPE_EAU=0; //

}

//FERMENTEUR 3

if(chrono_sec==1550-900){ //

POMPE_FERMENTEUR3=1; //

}

if(chrono_sec==1565-900){ //

POMPE_FERMENTEUR3=0;

}

if(chrono_sec==1580-900){ //

POMPE_EAU=1; //

}

if(chrono_sec==1610-900){ //

POMPE_FERMENTEUR3=1; //

}

if(chrono_sec==1615-900){ //

POMPE_FERMENTEUR3=0; //

}

if(chrono_sec==1620-900){ //

POMPE_EAU=0; //

GO_flag=0;

}

}// end go flag =1

if( GO_flag==0 && chrono_sec>900){ //

GO_flag=1;

chrono_sec=0;

chrono_synchro=0;

}

}// end while

}//end main

//------------------------------------------------------------------------------
