# Supplementary material for: A low-cost, multiplexable, automated flow cytometry procedure for the characterization of microbial stress dynamics in bioreactors
Source: Microb Cell Fact. 2013 Oct 31;12:100. doi: 10.1186/1475-2859-12-100 (PMC4228430; doi:10.1186/1475-2859-12-100)

**Supplementary file S4**

**Determination of the linear range of analysis of the accuri C6 flow cytometer**

The linear range of analysis of the accuri C6 flow cytometer has been determinated. Calibration has been performed by using 1 µm green fluorescent microsphere (Fluorosphere, Invitrogen)


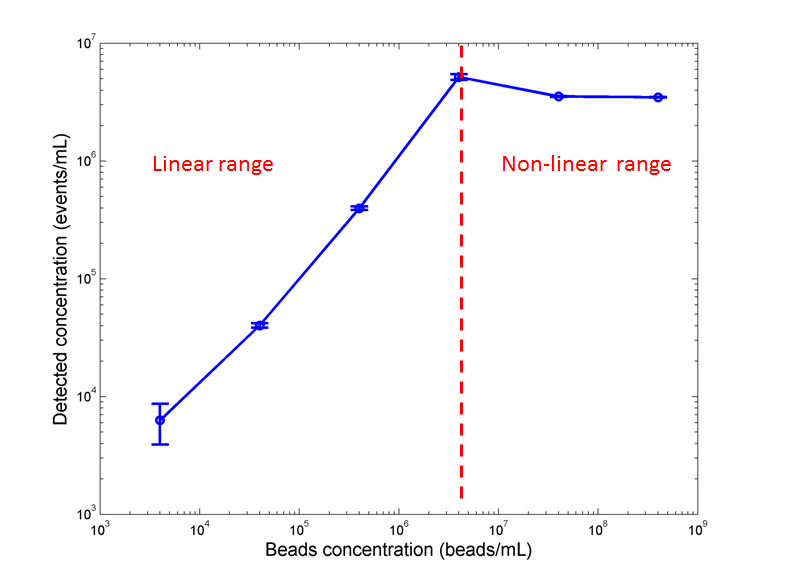

Supplement: Additional file 5: Figure S4 — Determination of the linear range of analysis of the accuri C6 flow cytometer. [file 1475-2859-12-100-S5.doc]
