# Supplementary material for: A low-cost, multiplexable, automated flow cytometry procedure for the characterization of microbial stress dynamics in bioreactors
Source: Microb Cell Fact. 2013 Oct 31;12:100. doi: 10.1186/1475-2859-12-100 (PMC4228430; doi:10.1186/1475-2859-12-100)
Supplement: Additional file 6: Figure S5 — Real-time monitoring of PI uptake and comparison with off-line staining. [file 1475-2859-12-100-S6.doc]

**Supplementary file S5**

**Real-time monitoring of PI uptake and comparison with off-line staining**

Microbial cells extracted at different phases of the bioreactor experiment are centrifuged and resuspended in phosphate buffer (pH 7) in Eppendorf tube (1 mL). The Eppendorf tube is placed in the sampling needle of the flow cytometer (FC) and the analysis is performed in continuous mode (sample is pumped into the FC at a flow rate of 14 µL/min). At the beginning of experiment, 10 µL of a propidium iodide (PI) solution (PI purchased from Sigma, 1 mg/mL in distilled water) is added directly in the Eppendorf tube during the FC analysis. Comparison with off-line staining is also provided. In this case, a contact time of 15 minutes, corresponding to the protocol often used in the literature, has been used.

A: cells extracted from a batch bioreactor during the exponential phase. PI is injected after approximately at the beginning of the real-time experiment. No PI uptake has been observed based on the FL3 signal. Off-lien staining have also been performed by considering different contact time between microbial cells and PI and the results are in good accordance with those obtained in real time B: cells extracted from a bioreactor during the transition from the batch phase to the chemostat phase at a dilution rate D = 0.14 h^-1^. In this case, two subpopulations can be observed after a contact time of 40 seconds. Anew, off-line analyses are in good accordance with those obtained in real time


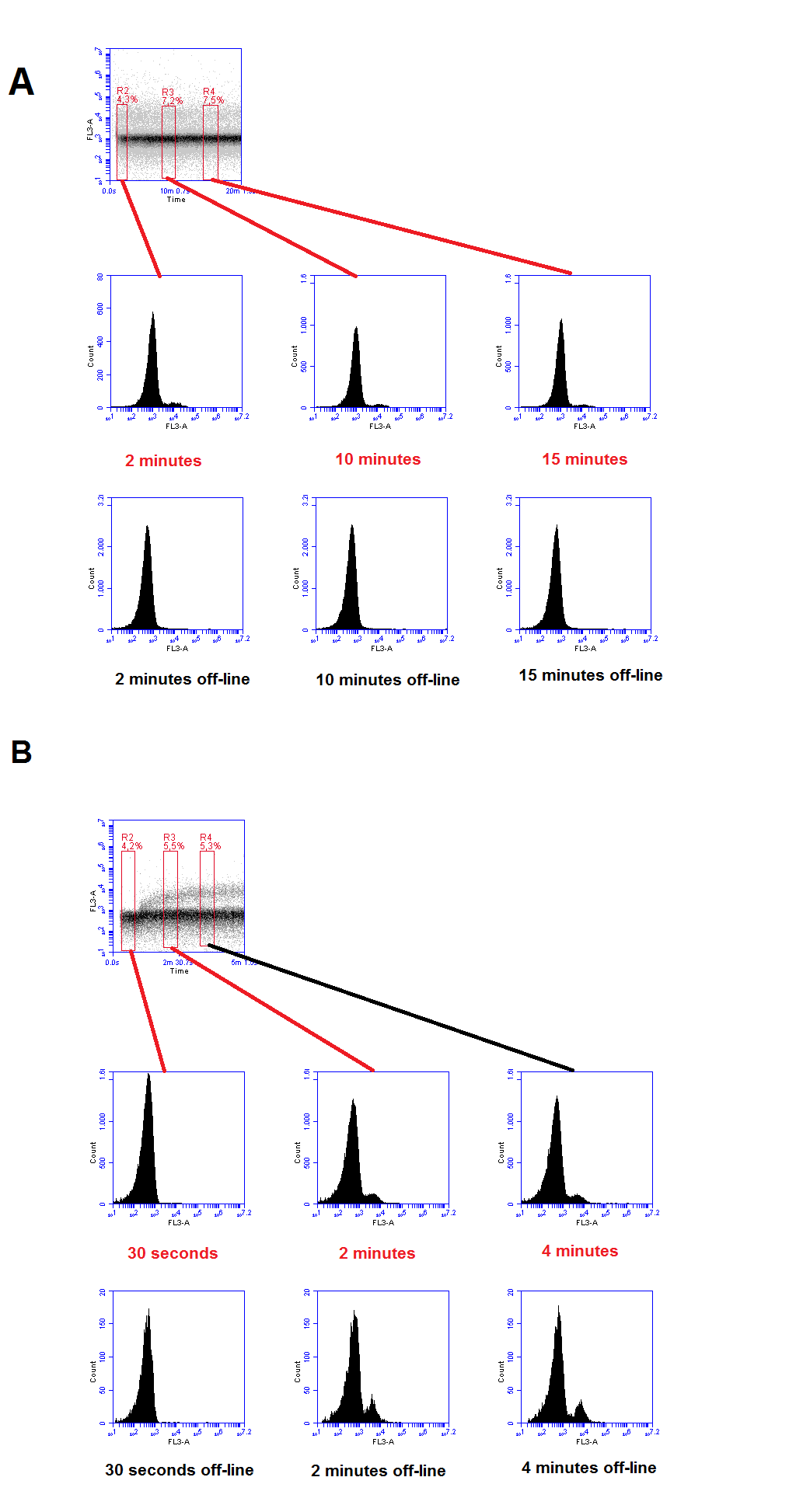


In our automated FC protocol, the contact time between cells and the dilution water containing PI has been deduced from these experiments and corresponds to 3 minutes.
